# Preparing for the next pandemic: predictors and effects of COVID-19 remote learning

**DOI:** 10.3389/fpsyg.2023.1256808

**Published:** 2024-02-13

**Authors:** Bodhi A. Brenner, Heather Thompson-Brenner

**Affiliations:** ^1^Cambridge Biotherapies, Cambridge, MA, United States; ^2^Boston University, Boston, MA, United States

**Keywords:** educational disparities, remote learning, distance learning, hybrid learning, standardized testing, COVID-19, charter schools

## Abstract

**Background:**

The COVID-19 pandemic forced school closures and rapid transitions to distance learning, which were widely associated with negative effects on educational attainment and mental health among youth. Research is now emerging about the relationship between distance learning and educational outcomes, as well as factors that sped or delayed the return of in-person learning in specific geographic regions. In the state of Massachusetts, in the United States, high schools (9th–12th grade) varied in the length of time that passed before in-person learning was offered. This study investigated (1) what factors were associated with the date at which schools implemented hybrid/in-person learning, and (2) what factors, including time in remote learning, were associated with loss of educational attainment.

**Methods:**

The sample included *N* = 267 regional/local high schools. Analyses investigated whether time to hybrid/in-person learning was associated with the percentage of students from low-income households and from minority ethnic/racial groups, local political affiliations and COVID incidence rate in September 2020, and the size of the district. The second set of analyses examined whether the high schools’ observed losses in standardized math test scores between 2019 and 2021 were associated with the amount of time students remained in exclusively remote learning, as well as the percentage of students from low-income households and minority ethnic/racial groups, the COVID cumulative incidence rate in the region by April 2021, and the size of the school district.

**Results:**

Multiple linear regression analysis examining variance in the date at which hybrid/in-person learning was implemented was most strongly predicted by the size of the school district. Multiple linear regression analysis examining variance in the loss of educational attainment was most strongly predicted by the percentage of students from low-income households in the high school. Exploratory analyses comparing charter schools with regional public high schools found that charter schools showed significantly greater loss of educational attainment, contrary to hypotheses.

**Conclusion:**

Additional protections for students from larger school districts, lower-income families, and charter schools are needed in case of future population-level disruptions in education.

## Introduction

The COVID-19 pandemic has been widely associated with negative effects on educational attainment and mental health among youth (e.g., Betthäuser et al., 2023). In the state of Massachusetts, in the United States of America, public school educational attainment dropped significantly between 2019 and 2021, as reflected in the standardized Massachusetts Comprehensive Assessment System (MCAS) scores administered annually to students in elementary and high schools across the state (Vaznis, 2021). An initial report on educational loss among elementary school students in the United States during the pandemic estimated the loss, on average, at the equivalent of 5 months of expected educational attainment (Dorn et al., 2020). The observed losses were not distributed equally across racial/ethnic groups; for example, African American children—who already demonstrated disadvantages in educational opportunity prior to the pandemic—were observed to have lost 6 months relative to pre-pandemic expectations, significantly more than other racial/ethnic groups (Dorn et al., 2020). The effects of educational loss and disruptions to school attendance may have far-reaching consequences. Educational loss is associated with reduced lifetime earnings as well as negative effects on mental and physical health (Burgess and Sievertsen, 2020; Dorn et al., 2020; Shen, 2020; Betthäuser et al., 2023).

In Massachusetts, during the summer and fall of 2020, individual school districts developed and implemented remote, hybrid, or in-person learning plans (with some state guidance), a logistical problem faced by educators all over the world (Chatzipanagiotou and Katsarou, 2023). Distance learning was implemented prior to September 2020, across all high schools in Massachusetts, but the decision to return to some form of in-person learning was made by individual school districts, balancing the importance of in-person learning along with the safety of students, families, and teachers. School committees had to consider multiple logistical factors specific to each district, including size, configuration, and ventilation of various school buildings; bussing patterns and resources; and the specific needs of different age groups. School districts also had to consider family preference, the political climate, teacher preference, and issues of equity and social justice (Chatzipanagiotou and Katsarou, 2023). Many school districts prioritized hybrid and in-person learning for students who were younger and those with specialized learning needs, phasing in high school hybrid education later in their back-to-school process. As a result, some 9th–12th graders in public high schools in Massachusetts returned to some version of in-person learning as soon as September, 2020, while others remained entirely remote until as late as September, 2021.

High school students polled during the pandemic in Massachusetts reported a preference for hybrid/in-person learning over remote learning, as well as the perception of educational loss and stress associated with remote learning (Gallup, 2021). In addition, poll respondents with lower family incomes were more likely to be in remote learning than those with higher family incomes (Gallup, 2021).

Preliminary analyses of the standardized Massachusetts Comprehensive Assessment System (MCAS) scores from Massachusetts after it was re-instituted in 2021 indicated that school districts with lower economic security and with higher minority enrollment suffered disproportionate loss in educational attainment in the period since 2019 relative to other groups (Vaznis, 2021). The specific association between educational loss and the protracted use of distance learning has not been demonstrated, however.

The MCAS assessment was first implemented in Massachusetts in 1993, as part of the Massachusetts Education Reform Act (MERA), which aimed to address inequalities in education. MERA increased the role of the state in funding under-performing schools, supported the formation charter schools that could serve local student needs, and created a system to identify the effects of interventions to address educational inequality, via the annual MCAS test. The reforms associated with MERA have been linked to outstanding educational performance in Massachusetts relative to other states, as well as reductions in the observed educational disadvantages experienced by students from minority racial/ethnic groups and from low income households in the period prior to the pandemic (Gass and Chieppo, 2013).

Inequities in educational opportunities are well documented in the United States. School districts that draw upon taxation of wealthier communities spend more money per student on education than less wealthy communities, and these differences in pupil expenditure have been directly related to inequities in learning (e.g., Baker and Levin, 2014). Though officials in the state of Massachusetts have attempted to address this problem, in part through MERA and the policies that followed from it, it was still the case in 2019 that in-district per pupil expenditure ranged from approximately $11,000 USD to approximately $30,000 USD (DESE, 2021). These inequities are as-yet unaddressed, and should be investigated relative to loss of educational attainment during the COVID-19 pandemic.

“Charter schools” are defined as schools that receive government funding (are not private schools), but operate according to a specific set of guidelines and objectives (a “charter”) that is separate from the public school system in the district where it is located. The charter schools in the Boston Public School system have demonstrated some success in raising educational attainment in the period prior to the pandemic; for example, students in Boston public schools who were offered charter-school placements have shown better educational attainment than matched peers who applied for but were not offered charter school placements via lottery (Cohodes, 2018). While charter schools have diverse goals, organizational structures, and educational methods, some researchers had high hopes that charter schools would be better able to respond to the COVID-19-related disruptions in education, due to their flexibility, cohesiveness, and relatively small size compared to regional public schools (Boast et al., 2020). Data concerning how charter schools’ observed educational attainment persisted or suffered during COVID-19 are still needed.

Recent studies have shown that political affiliation was strongly related to attitudes toward COVID-19 in the United States, including attitudes toward risk of contracting COVID-19 and preventative measures such as lock-downs, social distancing, and masking (e.g., Doherty et al., 2020; Kiviniemi et al., 2022). A nationwide study conducted in the summer of 2020, as school districts were trying to make decisions about the mode of learning to offer in the fall, specifically observed that political party affiliation was directly related to whether parents thought that schools should return to full in-person, full remote, or hybrid learning (Horowitz, 2020).

Detailed data concerning the factors that contributed to different school districts’ choices of mode-of-learning for high school students—such as community COVID prevalence rate, the financial resources of different districts, the political affiliations of the school community, and the school district size—are very much needed. Equally important are data concerning the effect of mode-of-learning in 2021 relative to other variables that may have independently predicted loss of educational attainment, such as the community prevalence rate of COVID-19, disparities in economic resources, and racial/ethnic minority group prevalence. Given that many of observed risk factors for educational inequity are intercorrelated—e.g., lower financial security is associated with minority ethnic/racial group status; individuals from minority groups are more likely to have lower financial security, and to live in urban centers with large school districts and where COVID rates were higher—careful analyses that control for inter-correlated factors are necessary to identify the most at-risk sub-populations, particularly to set up plans and structures that would prevent the same inequities in case of a future pandemic or similar population-level disruption.

The study investigated the following research questions.

(1) What factors influenced the point in the school year at which Massachusetts high schools returned to some form of in-person learning (hybrid/in-person) during the school year 2020–2021?

*Hypotheses:* The amount of time that passed before high schools offered hybrid/in-person education will be positively associated with the schools’ proportion of students from low-income households, the schools’ proportion of students from minority ethnic/racial backgrounds, the cumulative prevalence of COVID-19 in the schools’ communities in September 2020, the proportion of Democratic voters in the schools’ communities, and the size of the school district.

(2) What factors influenced the amount of educational loss recorded among high schools in Massachusetts between the MCAS administered in 2019 and the MCAS administered in 2021?

*Hypotheses:* The amount of 10th grade educational loss in math between 2019-2021 will be positively associated with the amount of time until schools offered hybrid/in-person learning in the 2020-2021 school year, the proportion of students in the schools from low-income households, the proportion of students in the schools from minority ethnic/racial backgrounds, the COVID-19 cumulative incidence in the school community in April 2021, and the size of the district (total enrollment) in which the schools were located.

(3) Were there differences in educational loss between charter schools and regional public high schools between 2019 and 2021?

*Hypotheses:* Charter schools will show lesser educational loss relative to regional public high schools, independently from the variance associated with the percentage of schools’ students from low-income and racial/ethnic minority groups.

## Methods

### Sample

The master dataset includes *N* = 347 public high schools in Massachusetts, including *N* = 267 regional and local high schools, *N* = 43 charter high schools, *N* = 35 Vocational/Technical high schools, and *N* = 2 completely virtual high schools. The MCAS test is administered to all 10th graders in public schools. Scores from every public high school in Massachusetts are included in the master dataset. Cases in the dataset represent schools. For reference, however, the total number of students enrolled in those schools in school year 2020–2021 was 288,358 and the total number of 10th graders was 72,925 (exact numbers varied slightly from 2019 to 2021). For Research Questions 1 and 2, we excluded charter, vocational/technical, and completely virtual schools, utilizing the *N* = 267 regional/local schools. The data were downloaded from the Department of Elementary and Secondary Schools (DESE) School and District Profile website.^1^ Data reflecting different levels of aggregation (e.g., school vs. district proportion of students from minority ethnic/racial backgrounds; individual school loss of educational attainment vs. school district size) were downloaded from different DESE reports and databases and then merged, matched, and cleaned. Other details regarding data collection are described in “Assessments” below.

## Assessments

### Massachusetts comprehensive assessment system: educational attainment in math

The MCAS was first implemented in Massachusetts public schools as part of the 1993 Massachusetts Educational Reform Act, which aimed to record and address educational inequality. Students in 10th grade are required to take the MCAS test, which includes Math, English Language, and Science sections, to assess school performance as well as demonstrate competencies to graduate from high school. School scores are compared across years by the Department of Elementary and Secondary School Education (DESE) to identify areas of inequality, schools that are not meeting benchmarks, and improvements across time in response to school and state efforts. Historically, Massachusetts has had an annual state participation rate over 98% across all grades, subjects, and assessments (Massachusetts Department of Elementary and Secondary Education, 2022; the reliability and validity of the items and tests are continually assessed and reported in great detail in the annual Next-Generation MCAS and MCAS-Alt Technical Report). For this study, the Math Scaled Score was chosen as the variable most representative of educational attainment achieved or lost in school across the time period, because English Language acquisition has been observed to have more varied external influences outside of school instruction, and Science education was noted to have been delivered more erratically than Math instruction during on-line and hybrid learning periods. The MCAS was administered to all 10th graders prior to the pandemic, in the year 2019, in the classroom according to strict protocols. In 2020 the MCAS was canceled. In 2021 it was again administered to the 10th grade students in accordance with the same protocols. Each high school’s overall (average) Math Scaled Score was downloaded from the (publicly accessible) DESE website. To ascertain the degree to which 10th graders in each high school suffered educational loss between 2019 and 2021, we calculated the difference in school performance in 2019 and 2021. The loss scores therefore show a larger negative number for more math loss, a smaller negative number for less math loss, and a positive number for gains in math educational attainment as recorded on the MCAS (additional information about the MCAS data, as well as scores and score reports, are available at the DESE website: https://profiles.doe.mass.edu/statereport/nextgenmcas.aspx).

### Mode-of-learning: month that hybrid/in-person learning was implemented

When we initially conceived of this project, we hoped to create a variable that would reflect how much in-person learning was provided to 10th graders in the school year 2020–2021. This proved to be a difficult task. School systems reported to the DESE their *intentions* in the summer of 2020, and the large majority of schools reported the intention to implement hybrid learning, in accordance with state recommendations. However, as the school committees began to meet and plan, many school districts changed tack in response to a large number of factors, some noted earlier in the Background. We therefore decided that the most reliable indicator of variability in in-person learning that we could feasibly collect was the month (September, 2020, through May, 2021, or later) that the specific high schools actually brought 10th graders back into the school building for hybrid learning. The research required to obtain reliable data concerning this variable was extensive. The research team studied school committee meeting minutes, high school social media accounts, and published articles from reputable media sources, as well as called and emailed high school and school department personnel. We were able to verify the month that high school students returned to school in-person (initially all in hybrid models) for *N* = 257 high schools. The month that the school returned high school students to some form of in-person learning was recoded as 1 = September 2020, 2 = October 2020, 3 = November 2020, 4 = December 2020, 5 = January 2021, 6 = February 2021, 7 = March 2021, 8 = April 2021, 9 = May 2021, and 10 = later than May 2021.

The month at which hybrid/in-person learning was implemented for the charter schools in Massachusetts proved too difficult to attain. The return to hybrid/in-person learning for the relatively small charter schools was almost never reported in the local media, their decision-making was not recorded in district-wide school committee meetings, and social media accounts for charter schools, if they existed, were generally not updated during this chaotic period. Extensive research (e.g., visits to each charter school and interviews with multiple school employees) would be needed to investigate this variable in charter schools in the future.

### Proportion of students from low-income families

This variable was downloaded from the DESE school profiles website. The percentage of students in each high school from low-income households was downloaded and merged with the individual high school MCAS data, and double-checked to make sure entries were correctly matched with schools.

### Proportion of students from minority/ethnic backgrounds

This variable was downloaded from the DESE school profiles website. The percentage of students from minority racial/ethnic backgrounds for each school was downloaded, summed across different minority racial/ethnic groups, and merged with the high school MCAS data. Data were double-checked to make sure percentages were correctly matched with schools.

### District per-pupil-expenditure

This variable was downloaded from the DESE district profiles resources. Because multiple schools were included in single districts, per-pupil in-district expenditure for different districts were matched with all the high schools within that district.

### District size (total enrollment of K-12 students in district)

This variable was downloaded from the DESE district profiles website resources. Because multiple schools were included in single districts, the district size was matched with all the schools within that district.

### COVID-19 cumulative incidence rate

The Massachusetts Department of Public Health posted and archived daily COVID-19 data throughout the timeframe assessed. Using the tables of town and city data that were provided, we calculated the cumulative COVID-19 incidence (cumulative incidence positive COVID-19 cases corrected by the population of every town/city population, as reported by the Department of Health) on September 1, 2020 (when mode-of-learning decisions were being made) and on April 1, 2021 (near the time that the second MCAS test was administered). We then identified the town/city in which every high school was located and entered the COVID-19 prevalence rate for those two dates.

Our preliminary examination of the population-corrected COVID-19 rate on September 1, 2020, showed minimal variation, with nearly all communities outside of Metro Boston showing essentially the same community prevalence. Therefore, we concluded we could not test the relationship between the 2020 COVID variable and others of interest. The cumulative COVID incidence by April 1, 2021, varied widely enough to be included in analyses.

### Political preferences

Though Massachusetts is a majority Democratic state, there is variation in the percentages of Democrats and Republicans in different cities and towns. For this study, we accessed records regarding the final percentages of voters who chose the Democratic candidate in the presidential election (Joseph Biden) and the Republican candidate (Donald Trump) in every city/town in Massachusetts in November of 2020 (Smith, 2020). We entered the percentage of Democratic voters in every city and town in which the high schools were located.

### Data analyses

Data analyses were conducted in SPSS (Statistical Package for the Social Sciences) version 28.0.1.1 (14). Analyses included descriptive data, bivariate (Pearson’s product–moment) correlations between variables, and linear multiple regression for multivariate analyses using the variables that were significant in bivariate analyses. We examined the variables for variation, normalcy/skewness, and kurtosis, and outliers in SPSS. No outliers were identified in the variables included in analyses of the time to hybrid/in-person learning. Repeated analysis of standardized residuals identified *N* = 9 outliers in the independent variable reflecting loss of educational attainment (MCAS math score change). These cases were removed from relevant analyses.

## Results 1: Factors associated with time until in-person (hybrid) learning was implemented

**Figure 1:**
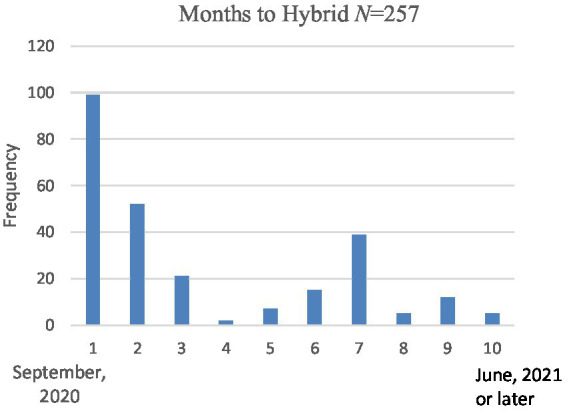
Number of high schools implementing hybrid learning by month.

For Research Question 1, which focuses on the factors related to the date that different schools implemented hybrid learning, the sample included *N* = 267 regional high schools. Of those schools, we were able to obtain reliable and verifiable information regarding date of first hybrid learning for *N* = 257. As Figure 1 shows, the largest proportion of schools implemented hybrid learning in September and October of 2020. There was another large proportion that implemented hybrid learning in March of 2021, primarily due to the fact that the entire Boston public school system (*n* = 25, or 9%) implemented hybrid learning at that time. Due to this observation, we conducted later correlational and multivariate analyses both with Boston schools and without Boston schools, to see if the results were skewed by this single district.

**Figure 2:**
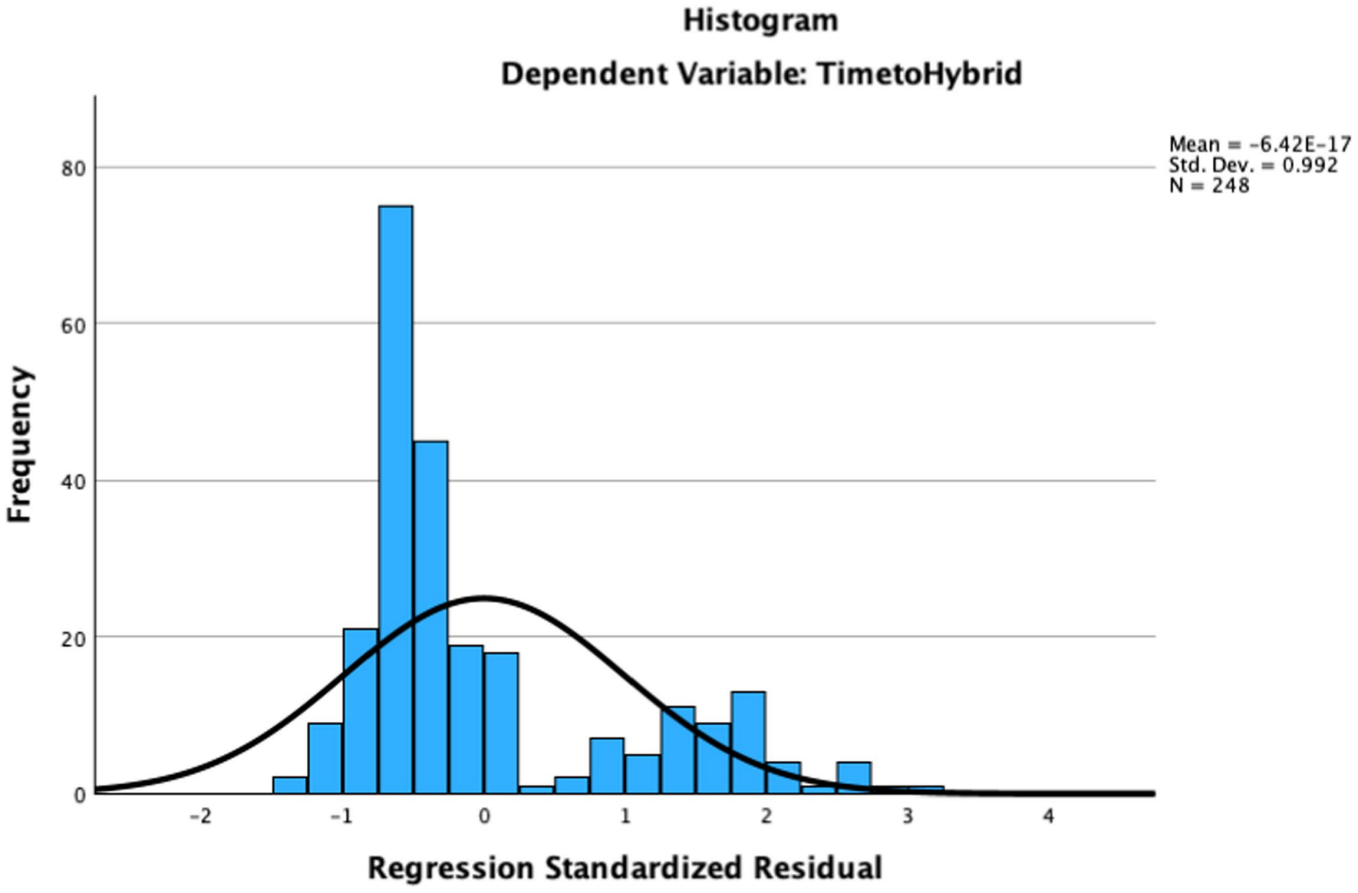
Standardized residuals for time to hybrid learning from linear regression model.

Each of the variables considered for inclusion in the analysis were examined for normality; results concerning skewness and kurtosis indicated the variables were in the acceptable range (skewness between −3 and 3, kurtosis between −10 and 10; Brown, 2006). While Figure 1 seems shows the appearance of a non-normal curve, this was due to the absence of schools implementing hybrid learning in December (just prior to the holiday vacation). In fact, the distribution as assessed statistically (see Figure 2) showed acceptable normality.

Table 1 shows the substantial variation in the percent of students in each school from minority ethnic/racial groups and from low-income households, as well as the district per pupil expenditure, and the size of the district in which the schools were located. The percentage of each city/town that voted for Biden ranged from 41.4 to 92.0%.

**Table 1:** Descriptive statistics and correlations with time to hybrid learning in regional public high schools in Massachusetts (*N* = 257 schools).

Variable	*M*	*SD*	r*	*p*
1. Percent of students from minority ethnic/racial groups-school	35.45	28.67	0.47	<0.001
2. Percent of students from low-income families-school	39.57	24.52	0.39	<0.001
4. Proportion voted Democrat-town/city	0.65	0.12	0.31	<0.001
5. Total pupils in school district	8,317	13,151	0.53	<0.001

*r*-value for correlation with the number of months until in-person/hybrid learning was implemented; *p*-value for statistical significance of the correlation.

Table 1 also displays correlations to assess the relationship between the month that hybrid learning was implemented and other variables of interest. As noted, we conducted analyses both with Boston data and without Boston data, because there were so many schools in the Boston district. Upon comparison, all analyses showed similar results with and without Boston data, with the exception of the correlation between the month that hybrid was implemented (which was decided at the district level) and per-pupil expenditure (which is calculated at the district level). We concluded that inclusion of Boston schools could skew this relationship because the district had a relatively high per-pupil expenditure ($25,217 per pupil), but was relatively late to implement hybrid learning (March, 2021). When we calculated the correlation between district per pupil expenditure district, and the month that hybrid learning was instituted in the district, without the Boston data, there was no correlation between these variables (*r =* −0.003, *p = 0*.485). Therefore, we concluded that the Per Pupil Expenditure variable was not of use in explaining the time until hybrid was implemented, and we should exclude it from further analyses.

As Table 1 shows, there were positive and highly significant relationships between each of the other demographic variables of interest and the amount of time that it took for hybrid learning to be implemented in the school. These correlations provide initial tentative support for the hypotheses associated with Research Question 1.

Next, we conducted multiple linear regression analysis to see which of the hypothesized variables were most strongly related to the time to implementation of hybrid learning.

Multiple regression analysis was conducted with the rating (1–10) of the month that hybrid learning was implemented as the dependent variable, with independent variables reflecting percent of students from minority ethnic/racial groups, percent of students from low-income households, percent of the town that voted for Biden in November 2020, and the size (total enrollment K-12) of the district in which the school was located. The results of the linear regression model were significant [*F*(4, 243) = 25.853, *p* < 0.001, *R*^2 =^ 0.282], explaining more than 25% of the total variance in amount of time that it took to implement hybrid learning. The only significant independent variable was the size of the school district, which significantly predicted Time to Hybrid [*B* = 0.45, *t*(243) = 5.003, *p* < 0.001].

Durbin-Watson test indicated the data met the assumption of independent errors (value = 1.769). Examination of the histogram of standardized residuals, scatterplot, and variance statistics suggested that the data displayed acceptable levels of normally distributed errors, homogeneity of variance, and linearity, respectively. Multicollinearity diagnostics, however, indicated a concerning level of multicollinearity present for the variable reflecting percentage of students in the school from ethnic/racial minority backgrounds (VIF = 6.37; Tolerance = 0.16), in part due to intercorrelations between this variable the percent of students from families with low SES. Neither of these independent variables was significant in this model, however, and removal of the variable reflecting percent of students from ethnic/racial minority backgrounds did not substantially change the results. We also conducted the same analysis excluding the Boston schools, to make sure that the large number of schools in that district did not skew the results, and results were also nearly the same.

The results of statistical analyses lent partial support to hypotheses associated with Research Question 1. In the multiple regression model, only the Size of the District (total enrollment K-12) predicted significant independent variance in the length of time in months until some form of in-person learning was implemented in regional public high schools (Table 2).

**Table 2:** Linear regression predicting amount of time to in-person/hybrid learning implementation (*N* = 248 schools).

Independent variables	B	SE	*t*	*p*
Intercept	1.985	1.186	1.674	0.095
Percentage students in minority ethnic/racial groups-school	0.000	0.013	−0.017	0.986
Percentage students in low-income families-school	0.010	0.012	0.905	0.366
Percent of democratic party voters-town/city	0.235	1.861	0.126	0.900
**Total pupils in school district**	**0.001**	**0.001**	**5.003**	**<0.001**

## Results 2: Factors associated with loss of educational attainment

Research Question 2 concerns the predictors of Loss of Educational Attainment between 2019 and 2021 among 10th graders in regional public high schools, reflected in the difference between 10th grade Math MCAS scores in 2019 and 2021. The descriptive statistics for this research question are displayed in Table 3. As Table 3 shows, the average math loss across regional high schools in the 10th grade was approximately five points on the Math MCAS 10th grade test, and the variation was substantial. The COVID-19 cumulative community incidence rate on April 1, 2021, ranged from approximately 0.02–2.24%.

**Table 3:** Descriptive statistics for 2021 change in math score and cumulative covid incidence and correlations (*N* = 258 schools).

Variable	*M*	*SD*
1. Change in 10th grade MCAS math score	−4.75	3.74
2. Cumulative covid incidence 4/1/21	0.08	0.03

**Figure 3:**
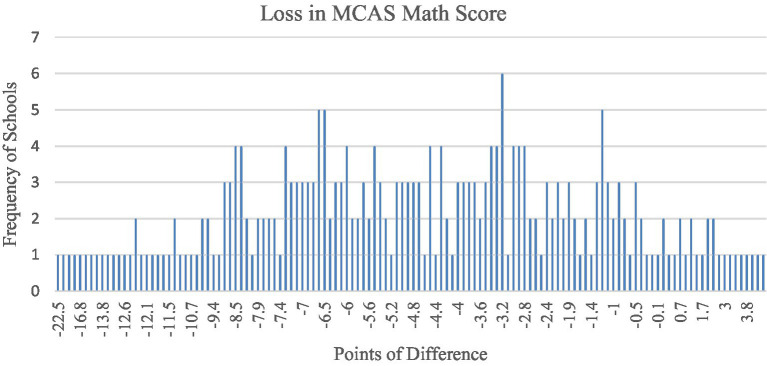
Bar graph of change in MCAS 10th grade math score between 2019 and 2021.

As Figure 3 shows, the amount of math loss was fairly normally distributed, with the majority of the schools in the curve losing between 9 points and 1 point on the MCAS 10th Grade Math Results, with two long tails. A small number of schools showed moderate *gains* in math score during the pandemic, and a slightly larger number of schools showing more dramatic decreases in scores from (from 10 to 22.5 points). As noted earlier, analysis of residuals indicated nine outliers in this variable, which were removed from analyses.

As with other analyses, we conducted bivariate correlations between the change in 10th grade MCAS Math score from 2019 to 2021 and variables of interest both with and without the Boston schools; the results of the analyses were virtually identical. The results of these analyses in the full sample (including Boston schools), but excluding outliers in educational loss in MCAS Math score, are displayed in Table 3.

As noted in the Methods section, a larger decrease in math attainment is reflected by a lower number (more negative change). The COVID-19 incidence, percentage of students from minority racial/ethnic background, percentage of students from low-income households, and the size (total enrollment) in the district are each scored on a positive scale, where higher numbers mean more of the respective variable. Therefore, negative correlations can be interpreted as the higher the independent variable, the more math loss observed in the school.

As Table 4 shows, the month at which hybrid learning was first offered showed a small but statistically significant correlation with Change in 10th Grade MCAS Math Score, as did the size of the district and the percent of ethnic/racial minority students in the school, and the cumulative COVID-19 incidence on April 1, 2021, suggesting higher values in these variables were associated with a small amount of loss in educational attainment. The percent of students in the school from low-income households, however, showed a larger and more significant relationship to loss of math attainment.

**Table 4:** Correlations with change in 10th grade math score 2019–2021.

Variable	*r*	*p*
1. Time to in-person/hybrid learning (*N* = 248)	−0.12	0.028
2. Cumulative covid incidence 4/1/21 (*N* = 258)	−0.22	<0.001
2. Percentage ethnic/racial minority students (*N* = 258)	−0.20	<0.001
2. Percentage low-income students (*N* = 258)	−0.34	<0.001
2. Total pupils in school district (*N* = 258)	−0.15	0.008

Significance tested 1-tailed in accordance with directional hypotheses.

We included all the variables that were significantly correlated with MCAS 10th Grade Math Score in the multiple linear regression model. The model predicting 10th Grade MCAS Math Change score included the following independent variables: length of time until hybrid learning was implemented, percentage of students from minority racial/ethnic groups, percentage of students from low-income households, size of the district (total enrollment K-12), and cumulative town/city COVID-19 incidence on April 1, 2021. Results from the Durbin-Watson test indicated the data met the assumption of independent errors (value = 2.10). Examination of the histogram of standardized residuals, scatterplot, and variance statistics indicated that the data displayed normally distributed errors, homogeneity of variance, and linearity, respectively. Multicollinearity diagnostics noted a smaller but somewhat concerning level of multicollinearity for the variable reflecting percentage of students in the school from ethnic/racial minority backgrounds (VIF = 5.77, tolerance = 0.17), however these values did not reach the threshold to necessitate removal from the model. No other problematic levels of multicollinearity were noted for variables in this model (no VIF values >4 or tolerance <0.25).

The results of the linear regression model were statistically significant [*F*(5, 242) = 7.848, *p* < 0.001, *R*^2 =^ 0.014]. As the results in Table 5 show, the independent variable most significantly associated with loss of educational attainment was the percentage of students in the school from low-income households, significantly predicted change in MCAS Math Score [*B* = −0.074, *t*(242) = −4.2838, *p* < 0.001]. Of interest, in the multiple regression model, the percentage of students from minority ethnic backgrounds in the school actually showed a small but statistically significant positive relationship to educational attainment, contrary to hypotheses (see Table 5). These results provide partial support for the hypotheses associated with Research Question 2, as well as one unexpected finding.

**Table 5:** Linear regression model predicting amount of 10th grade MCAS math loss (*N* = 247 schools).

	B	SE	*t*	*p*
Intercept	−2.074	0.645	−3.218	0.001
Time to in-person/hybrid learning	−0.049	0.095	−0.017	0.606
Cumulative COVID incidence 4/1/21	−14.308	9.583	−1.493	0.137
**Percent students in ethnic/racial minority groups**	**0.050**	**0.019**	**2.651**	**0.009**
**Percent of students in low-income families**	**−0.074**	**0.015**	**−4.838**	**<0.001**
Total pupils in the school district	−2.574E-5	0.000	−0.809	0.419

Independent variables significant at the *p* < 0.05 level are highlighted in bold.

## Results 3: Comparison of charter schools and regional public schools

Research Question 3 is exploratory, and was added after reviewing the literature on charter schools. In exploratory analyses, we examined whether there was a difference in loss of educational attainment between charter high schools and regional high schools as reflected by the difference between 10th grade MCAS Math Scores in 2019 and 2021.

To explore the hypotheses associated with Research Question 3, we analyzed data from a dataset with a larger number of schools—including both charter and regional high schools—but a smaller number of variables: only those that were accessible through the DESE MCAS website. As noted earlier, this dataset includes *N* = 347 public high schools in Massachusetts, including the *N* = 267 regional schools, *N* = 43 charter schools, *N* = 35 Vocational/Technical schools, and *N* = 2 completely virtual schools. Analyses focused on regional and charter schools, and are shown in Table 6.

**Table 6:** Regional and charter schools comparisons of means.

	Regional schools (*N* = 267)		Charter schools (*N* = 43)			
	*M*	*SD*	*M*	*SD*	*t*	*p*
10th grade MCAS math change	−5.00	4.44	−8.51	5.30	4.67	<0.001
Percent students minority race/ethnicity	35.37	28.65	68.26	29.27	−6.97	<0.001
Percent students low-income households	40.28	23.35	59.02	21.26	−4.89	<0.001

As Table 6 shows, charter high schools had a significantly larger mean decrease in 10th Grade MCAS Math Scores, a higher percentage of students from minority ethnic/racial groups, and a higher percentage of students from low-income households.

We next conducted a linear regression model assessing independent variance in MCAS 10th Grade Math Change score accounted for by the type of school (charter vs. regional, dummy coded), the percent minority students in the school, and percent of students from low-income households in the school. Results from the Durbin-Watson test indicated the data met the assumption of independent errors (value = 2.25). Examination of the histogram of standardized residuals, scatterplot, and variance statistics indicated that the data displayed normally distributed errors, homogeneity of variance, and linearity, respectively. No problematic levels of multicollinearity were noted for variables in this model (no VIF values >4 or tolerance <0.25).

The results of the linear regression model were statistically significant [*F*(3, 304) = 15.593, *p* < 0.001, *R*^2 =^ 0.133]. As Table 7 shows, two significant independent variables predicted loss in 10th Grade MCAS Math Score: charter schools were associated with greater loss, and the percentage of students in the school from low-income households were associated with greater loss.

**Table 7:** Linear regression model examining 10th grade MCAS math loss in charter vs. regional schools (*N* = 308 schools).

	B	SE	*t*	*p*
Intercept	−2.829	0.515	−5.490	<0.001
**School type (0 = regional, 1 = charter)**	**−2.55**	**0.783**	**−3.253**	**0.001**
**Percent students in low-income families**	**−0.065**	**0.016**	**−3.963**	**<0.001**
Percent students in minority ethnic/racial groups	0.012	0.013	0.922	0.357

Independent variables significant at the *p* < 0.05 level are highlighted in bold.

These results were unexpected, as the tentative hypothesis regarding charter schools was that they would be better able to respond to the stresses of COVID-19 learning, and show lesser loss in educational attainment. However, charter schools showed more loss in educational attainment during the pandemic, independently of the variance accounted for by the larger proportion of students from minority ethnic/racial groups and low-income households in charter schools.

## Discussion

The first part of this study aimed to investigate the factors associated with the length of time that it took for public high schools in Massachusetts to implement some form of in-person/hybrid learning during the school year 2020–2021, during the COVID-19 pandemic. We found that the majority of regional public high schools, nearly 60%, implemented hybrid learning in September or October of 2020, with the remaining 40% varying widely over the next 8 months. We found that the month in which hybrid learning was implemented was significantly correlated with several hypothesized factors, including the size (total enrollment) of the school district in which the high school was located, the proportion of high school students in the high school from low-income households, the proportion of students in the high school from minority racial/ethnic backgrounds, and the percentage of voters in that region who chose the Democratic candidate in the 2020 presidential election. We were not able to assess the effect of community incidence of COVID-19 in September of 2020, because the community incidence rate on that date was consistent across all communities outside the Metro Boston region. These various demographic factors were highly inter-correlated. When all the significant factors were entered into an multiple regression model, only the size of the district (total enrollment K-12) remained significant, accounting for a quarter of the variance in the length of time to hybrid implementation in regional high schools.

Larger school districts faced far greater logistical challenges than smaller school districts when attempting to implement hybrid learning (Chatzipanagiotou and Katsarou, 2023). The smallest school districts in Massachusetts had less than 500 students, whereas the largest school district had over 45,000 students. The Boston public school system had to juggle the concerns of thousands of children in hundreds of elementary and high schools—including bussing, teaching, air quality, and social distancing—whereas smaller districts had to contend with only a handful of schools. However, in Massachusetts, larger district size is associated with urban centers—such as Boston, Worcester, Springfield, and Framingham—which have larger proportions of students from minority racial/ethnic groups and from low-income households. The inequality that is associated with receiving an education in a large, urban public school system has been the focus of Massachusetts school reform for many years, with some demonstrated success (Gass and Chieppo, 2013). However, the effects of the COVID-19 pandemic reveal the structural inequalities and vulnerabilities associated with school districting.

The inequalities that follow from regional school districting that disproportionately affect low-income families have been the focus of research and policy attention nationally (e.g., Saiger, 2010; Harris, 2017). Some efforts to address the problem have included interdistrict enrollment plans, which allow students to register for schools outside their district, typically by application and if they demonstrate membership in a minority ethnic/racial group or lower socioeconomic status (Harris, 2017). These plans have not had widespread success, due to the limited effect of selective admissions, the inconvenience and negative social experience of out-of-neighborhood schooling, and the many incentives for wealthier communities to push against these policies. Other limited programs, such as charter schools, magnet schools, and school vouchers, have also not yielded widespread effects, particularly in addressing the structural inequalities (Harris, 2017). Many school reforms implemented in the last two decades, including in Massachusetts, have focused on identifying and raising the standards of underperforming schools and districts to an “adequate” level, with some measure of success. However, the widespread practice in the United States of delegating education to local school committees, rather than disestablishing districts and implementing federal and state control over schools (more common in Europe; Saiger, 1999), remains relatively stable and continues to demonstrate and support inequity.

This study also aimed to investigate whether longer time in remote learning was associated with loss of educational attainment among Massachusetts high school students, as reflected in the difference in 10th Grade MCAS Math scores between the 2019 and 2021. This study found a small but statistically significant association between longer time in all-remote schooling and loss of educational attainment. In multiple regression models that included the amount of time in all-remote learning along with local COVD incidence, percent of students from minority racial/ethnic backgrounds, and percent of students from low-income families, only the proportion of students in the school from low-income households was significantly related to school loss of math educational attainment.

The field of research on the topic of remote learning has burgeoned in the few years since remote learning was rapidly and widely implemented due to COVID-19. Some of this research has demonstrated negative effects associated with remote learning: For example, research on secondary school education in Brazil suggested that remote education was associated with much elevated drop-out rates and lower test scores, and these negative indicators were largely redressed when in-school learning was offered again (Lichand et al., 2022). Other research has investigated factors associated with variation in the quality of remote learning that was provided during the pandemic. For example, research with college-aged students in Spain, utilizing structural equation modeling, indicated that, while college students had a generally negative opinion of remote learning, the quality of the teacher-student interactions and the quality of materials provided to the students predicted higher opinions of remote learning among the students (del Arco et al., 2021). This research is still emerging, and conclusions about these effects and future recommendations for high schools education in particular have yet to converge.

Nationwide research on educational loss in the United States across elementary and high schools has suggested extensive state-by-state variation (Bryant et al., 2023). One comprehensive study indicated an average four point/12 week learning delay nationally, but some states showed as much as a year’s delay, while other states’ students were only delayed by the equivalent of a few weeks (Bryant et al., 2023). This study found a weak but significant association between states’ remote vs. in-person learning policies and the amount of educational loss. The study observed that remote education policies may have had some effect on student achievement, along with other important factors such as pre-pandemic learning trends; the quality and effective implementation of remote-learning programs, other COVID-19 policies such as quarantine and testing, and the nature and quality of hybrid/in-person learning and active educational recovery efforts that were delivered subsequently (Bryant et al., 2023). The effect of remote learning on student achievement may vary according to age, as well, and specific research on high school remote learning is needed. Bryant and colleagues’ report concludes that states may redress these losses by accessing and targeting national Elementary and Secondary School Emergency Relief funds (2023).

The major finding from this study concerning educational loss supported the conclusion that across districts and school systems, the proportion of students from families with lower incomes showed the strongest association to educational loss among students in high schools. Families with greater financial resources can protect their children attending public high schools from loss of educational attainment when school systems are disrupted in multiple ways. Even within stressed urban high schools, the children in families with more economic means may have entered high school from local neighborhood schools with more resources, as well as hired tutors or involved family members in supplementing on-line learning during the pandemic (Sonnenschein et al., 2021; Haelermans et al., 2022). The parents in privileged families are more likely to work remotely, or to take paid leave, and therefore suffered less disruption. Families with more economic means could to invest in expensive high-speed WIFI connections, and trouble-shoot problems with technology in remote learning as they came up (Haelermans et al., 2022). Families from lower socioeconomic groups and minority racial/ethnic groups were less likely to have family members who contracted or died from COVID-19 during that time (Webb Hooper et al., 2020), or suffered a wide range of mental health and stress challenges during the epidemic, a burden suffered unequally by more and less privileged families within the same public high school learning environment. The COVID-19 inequalities suffered by low-income families highlight a terrible inequality that only grew greater at the time of population-level crisis.

This project also aimed to explore whether the charter school movement, which has been supported in Massachusetts in part to address educational inequality, showed promise to protect against disruptions in educational attainment during situations such as COVID-19. Our exploratory investigations found that contrary to expectation, charter schools sustained larger losses in educational attainment than regional schools during COVID-19, even when the proportion of students from low-income households and minority racial/ethnic groups were controlled. Nationwide research on charter schools in the United States during the pandemic has indicated that charter schools were more likely to maintain all-remote learning for longer periods of time (Petrelli, 2022). Further research is needed to understand why charter schools suffered disproportionate loss of educational attainment during COVID-19. Reasonable hypotheses include the possibility that such small, self-contained organizations, while possibly better able to respond to their students’ needs under regular circumstances, were less resourced, less regulated by state Departments of Education, and therefore and more vulnerable in the context of a population-wide crisis (Haelermans et al., 2022). Further research is needed to investigate the difficulties that charter schools had in serving their protective function during this crisis, to prevent such disruptions to already vulnerable students in the future.

### Limitations

There are many limitations to this study. The measurement methods we used for our key variables all had limitations.

- It was remarkably difficult to measure the amount of in-person schooling that was provided to high-school students in 2020–2021. This is understandable given the chaos that the public school system faced, nonetheless, our measure of in-person learning—the date that hybrid was offered to high school students—is extremely limited. In the future, if school is disrupted, it would be helpful to have a real-time tracking system for grades and schools to understand the effects of in-person, hybrid, and remote learning. This variable also does not reflect in any way the quality of the education, such as the amount of interactive learning included in the distance model, which may vary substantially.
- The difference between MCAS Math 10th grade scores in 2019 and 2021, assessed at the school level, is only a very rough measure of educational attainment loss. There are many well-known limitations to standardized testing, particularly for minority ethnic/racial groups, however, standardized testing in Massachusetts was implemented partially in order to provide an assessment that could help to identify and redress educational inequality, and provided useful to investigate inequality during this population-wide disruption in education.
- Percentage of the population in each town-city who voted for the Democratic presidential candidate in the November, 2020 election is only a rough measure of political attitudes, and furthermore, in Massachusetts, no district measured below 40% Biden voters. In other states, the political affiliations of the school board members, teachers, or parents may have been more influential on schooling decisions than we were able to show in Massachusetts.

In addition, there are many factors that may have had influence on the mode-of-learning and loss of educational attainment variables that were not assessed in this study. Through our investigation of school committee meeting notes, we observed that teacher preferences, as well as collective teacher union bargaining agreements, had some measure of influence that we were not able to measure. We also observed that certain school systems, generally with higher proportion of low-income and minority ethnic/racial students, were concerned with issues of equity that in fact may have influenced them to delay in-person learning, because of their concern that low-income and minority families would suffer from between-family differences more in in-person learning (for example due to higher rates of COVID-19, or lower vaccination rates) than they would in remote learning. Qualitative research is needed to understand how school committees in more and less advantaged communities made such decisions.

## Conclusion

The COVID-19 pandemic resulted in extreme educational disruption and loss in educational attainment among youth in the United States, including students in public high schools in Massachusetts. The amount of time that it took to provide in-person (hybrid) education in high schools in Massachusetts was strongly associated with the size of the school district, and the educational loss represented by loss in 10th grade MCAS scores between 2019 and 2021 was strongly accounted for by the proportion of students in high schools who came from low-income households. Charter high schools in Massachusetts, which were historically created in part to redress inequalities in education, did not show expected protection against educational attainment loss, but rather showed more loss, independent of economic and racial/ethnic factors. Though Massachusetts has been a leader in the United States in the movement toward educational equality, this study’s findings regarding the inequalities in the educational attainment associated with the COVID-19 pandemic in high schools in Massachusetts highlights the need for further research and further action to advance the aims of educational justice, particularly in the occurrence of population-wide disturbances.

## Data availability statement

The raw data supporting the conclusions of this article will be made available by the authors, without undue reservation.

## Ethics statement

Ethical review and approval was not required for the study on human participants in accordance with the local legislation and institutional requirements. Written informed consent from the patients/participants or patients/participants legal guardian/next of kin was not required to participate in this study in accordance with the national legislation and the institutional requirements.

## Author contributions

BB: Conceptualization, Data curation, Formal analysis, Investigation, Methodology, Visualization, Writing – original draft, Writing – review & editing. HT-B: Conceptualization, Formal analysis, Investigation, Methodology, Project administration, Supervision, Visualization, Writing – original draft, Writing – review & editing.

## Footnotes

^1^ https://profiles.doe.mass.edu
